# Expression of Concern: Coordination of agricultural informatization and agricultural economy development: A panel data analysis from Shandong Province, China

**DOI:** 10.1371/journal.pone.0362715

**Published:** 2026-11-13

**Authors:** The PLOS One Editors

After this article [1] was published, concerns were noted that the articles cited as References 4, 10, 36, 38, and 43 were retracted after [1] was published.

In light of the issues, the *PLOS One* Editors issue this Expression of Concern. Readers are advised to interpret the article [1] with caution.
