# Supplementary material for: Coordination of agricultural informatization and agricultural economy development: A panel data analysis from Shandong Province, China
Source: PLoS One. 2022 Sep 16;17(9):e0273110. doi: 10.1371/journal.pone.0273110 (PMC9481045; doi:10.1371/journal.pone.0273110)
Supplement: Attachment — Submitted filename: Response to Reviewers.docx [file pone.0273110.s003.docx]

**I suggest to improve literature source with international units.**

**Reviewer #1: The topic of the paper is relevant and very interesting. Authors used both national and international literature sources and cited them correctly. Most part of them are from the last 10 years. I suggest to improve literature source with international units. I agree that the continuous development of the world economy, science, and technology, the era of intelligence and information is upon us, which is true all sectors, such as the agriculture. Authors chose a major agricultural province in China, Shandong Province which has been ranked first in China for many years in terms of gross agricultural product and the import and export of agricultural products. This paper uses evaluation index data of Shandong Province from 2011 to 2019. Authors used different – relevant – methods in their research work. They presented their results and introduced them in a well-structured form using tables and figures. I appreciate their conclusions and agree those limitations which they wrote at the end of their research work.**

*Thank you very much for your valuable suggestion. Based on your suggestion, I have added 12 international literature sources.*

**Reviewer #2: Comments and Suggestions for Authors
Thank you for the opportunity to read the study paper. Topic is very interesting and topical. Topicality is obviously supported by the fact of agricultural informatization and its strong relation with agricultural economy development.
Title: The title is a little too long, but acceptable to me.**
*Thank you very much for your valuable suggestion.*

**1. Abstract and introduction is well-structured and clear at the present form but the relevance and significance and novelty value of the entropy value method and coupling coordination degree analysis are missing. It should be more focused on the importance of the topic or main issue and gaps more.
The literature review. The literature review section (combined from introduction and overview of the study area) is good and well balanced.**

*The relevance between them was added to section 4.2, and innovation and research significance were added to section 4.1.*

**2. Please correct or explain the followings:
A reference should be made to the table 2.1. In addition, all tables require resources, even if they are reconstructed or modified. What is the source of table 3.1?**

*I added the source of literature to all the tables, thank you.*
**3. Studying and interpret of table 3.2 is very difficult in the form of simple data, it is hard for humans, it is not communicate well, so a graphical representation of the data is necessary to draw further conclusions. A graph figure should be done it.**
*I have added another chart as Figure 3.1 in the corresponding position. The table and the chart can be seen more clearly together. Thank you.*

**4. In the line 155 and 156: instead of capital ‘I’ use ‘i’
please harmonize ith; jth or i-th; j-th or JTH (My suggestion: ith or jth)
In the line 176: where n is the number of years
In the line 177: please explain “information entropy” or make a reference for it
In the line 185: n and k is the same, so use n instead
In the line 190: Ui where Ui represents the indicator in the i-th year (if I’m right or make it clear)
In table 4.1 I recommend the same or similar expressions if possible on both side (minus and plus). The ‘primary coordination’ is unclear (I suggest ‘mild coordination’) … moderate coordination, good or serious coordination, high-quality coordination**

*I sincerely apologize for the mistake. I have made some modifications to solve these problems.*

*Because these are coordination levels, I looked up some literature and found that they are not plus or minus signs. Thank you very much for your valuable suggestion.*

**5. Discussion and enlightenment – This part is very good, but most of the findings seem to me to be a strategic program for the government or municipalities, not implications. Based on this, I suggest focusing more where possible on specific, short-term actions rather than a long-term focus or concept. (Although trainings and awareness raising are definitely good options.) If possible, also formulate generalized results that are not concerning to Shandong Province only.**

*I have deleted “Give full play to the principal role of the government” and added new content, “Strengthen the integration of information technology and agriculture with regional characteristics”.*
**Research findings (Conclusions and limitations) – These section is appropriate.
The paper has very good potential, but the authors need to make a minor revision at this stage. I’m very glad to have the opportunity to read your work.**

*Thank you very much.*
